# Schizophrenia is not associated with the *ERBB3* gene in a Han Chinese population sample: Results from case-control and family-based studies

**DOI:** 10.1590/S1415-47572009005000088

**Published:** 2009-12-01

**Authors:** Dawei Li, Guang He, Yifeng Xu, Yun Duan, Niufan Gu, Xingwang Li, Yongyong Shi, Wei Qin, Guoyin Feng, Lin He

**Affiliations:** 1 Bio-X Center, Key Laboratory for the Genetics of Developmental and Neuropsychiatric Disorders, Shanghai Jiao Tong University, ShanghaiChina; 2 Institutes of Biomedical Sciences, Fudan University, ShanghaiChina; 3 Shanghai Institute of Mental Health, ShanghaiChina; 4 Institute for Nutritional Sciences, Shanghai Institute of Biological Sciences, Chinese Academy of Sciences, ShanghaiChina

**Keywords:** schizophrenia, *ERBB3* gene

## Abstract

*ERBB3* (v-erb-b2 erythroblastic leukemia viral oncogene homolog 3), encoding a receptor of neuregulin-1 (NRG1), has been considered a functional candidate gene for schizophrenia susceptibility. In order to investigate a relationship between *ERBB3* gene and schizophrenia in the Chinese population, case-control and family-based studies were carried out in 470 cases matched by controls, and in 532 family trios. Our results failed to show any evidence of significant association between the *ERBB3* rs2292238 polymorphism and schizophrenia.

Evidence of association between the *NRG1* (Neuregulin-1) gene and schizophrenia has been documented (Li *et al.*, 2006). The association of schizophrenia and single-nucleotide polymorphisms (SNPs) of *ERBB4 (*v-erb-b2 erythroblastic leukemia viral oncogene homolog 4), enconding one of the receptors for NRG1 has been shown, thus suggesting that NRG1-ERBB signaling is involved in the pathogenesis of schizophrenia (Benzel *et al.*, 2007). ERBB3 is another NRG1 receptor, and its mRNA has been reported to be down-regulated in brains from schizophrenic patients (Hakak *et al.*, 2001; Tkachev *et al.*, 2003). These findings made *ERBB3* a functional candidate gene for susceptibility to schizophrenia. However, studies in samples from the Japanese population did not reveal any association between *ERBB3* polymorphisms and schizophrenia (Kanazawa *et al.*, 2007; Watanabe *et al.*, 2007).

In order to investigate the relationship between the *ERBB3* gene and schizophrenia in the Chinese population, we carried out case-control and family-based studies of the *ERBB3* rs2292238 polymorphism, using samples consisting of 470 cases matched by controls and 532 family trios (composed each one by normal parents and a single affected offspring), all of Han Chinese origin. The patients (mean duration of illness: five years) were interviewed and diagnosed according to the DSM-IV, by two independent psychiatrists. The case sample included 250 patients from northern and 220 from southern China; 227 (48.3%) were males and 243 (51.7%) females, with an overall mean age of 32.5 years (sd = 11.0). The controls consisted of 334 subjects from northern and 136 from southern China; 225 (47.9%) were males and 245 (52.1%) females, with an overall mean age of 32.6 years (sd = 9.5). The family trios were composed respectively by 170, 191, and 171 nuclear families from Shanghai, Anhui and Changchun; among the 532 probands, 260 (48.9%) were males and 272 (51.1%) females. A standard informed consent for the genetic analysis, reviewed and approved by the Shanghai Ethical Committee of Human Genetics, was obtained from all subjects.

Genomic DNA was extracted from peripheral whole blood using a modified phenol-chloroform method (Gao *et al.*, 2001). Real-time quantitative PCR with allele-specific amplification was performed through two PCR reactions for each sample, carried out in a total volume of 5 μL containing 10 ng genomic DNA, 2.5 μL Taqman® Universal PCR Master Mix (Applied Biosystems), 0.2 μM allele-specific primer, 0.2 μM common primer and 0.2x SYBR® Green I (Molecular Probes) on an ABI PRISM 7900 Sequence Detection System (Applied Biosystems). The primers used were: 5'-CAAAGTGTTGGGTAATTAGAAG T-3',5'-AAAGTGTTGGGTAATTAGAAGG-3', and 5'-TACCAGTTGGAACACTTAATCGG-3'. Protocols were as described by Liu *et al.* (2005).

A Monte Carlo approach (Sham and Curtis, 1995) was used in the case-control analysis with 100,000 simulations, in order to estimate exact test probability values. Transmission (TDT) and pedigree (PDT) disequilibrium tests, and haplotype-based haplotype relative risk (HHRR) analyses were used in the family-based samples.

We did not find any difference between cases and controls when genotype or allele frequencies were compared (χ^2^ = 1.188; 2 d.f.; p = 0.552 and χ^2^ = 1.130; 1 d.f.; p = 0.288, respectively). The results summarized in Tables 1  and 2 showed no evidence of significant association for rs2292238 using any of the above strategies. Furthermore, no evidence of association was found when the probands of the family trios were added to the affected sample of the case-control study (data not shown). The sample sizes we could get in this study would have a sufficient statistical power to detect the presence of a significant association, if present.

## Figures and Tables

**Table 1:** Results of case-control analyses.

	χ^2^ tests for HWE	Observed numbers and frequencies of genotypes				p values, Odds ratios, 95% CI			Observed numbers and frequencies of alleles			p values, Odds ratios, 95% CI
		GG	GT	TT		GG+GT *vs.* TT	GG *vs.* GT+TT		G	T		
Patients	0.2818 p = 0.515	52 0.110	216 0.461	201 0.429		0.389 0.89 (0.69-1.15)	0.367 0.83 (0.56-1.24)		320 0.341	618 0.659		0.288 0.90 (0.75-1.09)
Controls	0.0721 p = 0.788	61 0.130	220 0.469	188 0.401					342 0.365	596 0.635		

**Table 2:** Results of family-based analyses.

TDT			HHRR			PDT	
T = 220 NT = 237	χ^2^ = 0.632 p = 0.426		T = 220 NT = 237	χ^2^ = 0.632 p = 0.426		fr = 0.356	χ^2^ = 0.630 p = 0.427

TDT,Transmission disequilibrium test; HHRR, haplotype-based haplotype relative risk; PDT, pedigree disequilibrium test; T, number of transmissions, NT, number of non-transmissions.
